# Modifiable risk factors that mediate the effect of educational attainment on the risk of stroke: a network Mendelian randomization study

**DOI:** 10.1186/s13041-023-01030-0

**Published:** 2023-05-11

**Authors:** Bangbei Wan, Ning Ma, Zhi Zhou, Weiying Lu

**Affiliations:** 1 Reproductive Medical Center, Hainan Women and Children’s Medical Center, Haikou, China; 2 Central South University Xiangya School of Medicine Affiliated Haikou Hospital, Haikou, China

**Keywords:** Stroke, Educational attainment, Mendelian randomization, Proportion mediated, Genome-wide association study, Single-nucleotide polymorphisms

## Abstract

**Background:**

Stroke is a common cerebrovascular disease with great danger to public health. Educational inequality is a universal issue that influences populations’ stroke risk. This study aimed to investigate the causal relationship between education and stroke risk and the contributions of effects mediated by four modifiable factors.

**Materials and methods:**

Public large-scale genome-wide association study (GWAS) summary data associated with educational attainment, hypertensive diseases, body mass index (BMI), smoking behavior, time spent on watching the television (TV), and stroke were obtained from European ancestry. The data were used to investigate the causal relationship among educational attainment, hypertensive disease, BMI, smoking, watching TV, and stroke risk. Inverse variance weighted (IVW) method was used as a primary algorithm for estimating causal direction and effect size in univariable and multivariable Mendelian randomization (MR) analyses.

**Results:**

Higher educational attainment was a causal protective factor, while hypertensive diseases, higher BMI, smoking, and longer time spent on watching the TV were all causal risk factors for the risk of stroke. Hypertensive disease, BMI, smoking, and watching TV were all mediators for linking the causal relationship between educational attainment and stroke risk. Hypertensive disease, BMI, smoking, and watching TV explained 47.35%, 24.74%, 15.72%, and 2.29% of the variance in educational attainment’s effect on stroke risk, respectively. The explained proportion reached 69.32% after integrating the four factors.

**Conclusions:**

These findings support the causal effect of educational attainment on the risk of stroke, with a substantial proportion mediated by modifiable risk factors. Interventions on these modifiable factors would lead to substantial reductions in stroke cases attributable to educational inequality.

**Supplementary Information:**

The online version contains supplementary material available at 10.1186/s13041-023-01030-0.

## Introduction

Stroke is a common cerebrovascular disease with high mortality and disability [1]. In the past decade, stroke has mainly occurred in aging populations, and the incidence of stroke increases annually in young folks [2]. Although the incidence of stroke varies across different countries and regions, the number of patients with stroke is high. For example, observational data indicated that greater than 800,000 individuals are affected by stroke yearly in the United States [3]. In China, the populations affected by stroke are prominent, with more than 2 million people affected each year [4]. Additionally, the new stroke number in European people exceeded 1.1 million per year [5]. Considering the dangers of stroke, mapping out public policies to prevent stroke is necessary and urgent.

The educational level plays a vital role in the incidence of stroke. A recent investigation indicated that individuals with higher educational attainment have a lower risk of stroke than those with lower educational attainment [6]. A prospective cohort study revealed that individuals’ educational level was negatively correlated with their risk of stroke [7]. However, the educational level difference in global populations is a universal phenomenon and is difficult to change. Therefore, modifiable mediating factors of educational level influence on the risk of stroke need to be determined to prevent stroke. Stroke is a complex and multi-factorial disease. Many modifiable factors, including some diseases (obesity, hypertension, and diabetes mellitus) and lifestyle factors (tobacco smoking, physical inactivity, and poor diet or nutrition), are closely associated with the risk of stroke [8, 9]. Although factors such as obesity, hypertension, smoking, physical inactivity, and low educational level are positively associated with the risk of stroke [10–13], the causal inference of their underlying influence on the risk of stroke by interaction remains unclear. Whether educational level affects the risk of stroke mediated by the factors (obesity, hypertension, smoking, and physical inactivity) still needs further explanation.

Mendelian randomization (MR) study is a vital method of causal inference by using single-nucleotide polymorphisms (SNP) from genome-wide association study (GWAS) data as instrumental variables to estimate the causality between exposure(s) of interest and outcome(s) of interest [14, 15]. In the present work, we assessed the role of four modifiable factors, namely, hypertensive diseases, body mass index (BMI), smoking behavior, and time spent on watching the television, in mediating the causal effect of educational attainment on the risk of stroke by using MR. The inverse variance weighted (IVW) approach was used as the primary causal estimation method. Considering the crucial role of educational attainment, hypertensive diseases, BMI, smoking behavior, and time spent on watching the TV on the occurrence of stroke, further understanding the potential mechanism is beneficial in drawing up public health policy for preventing stroke.

## Materials and methods

### Overall study design

**Fig. 1:**
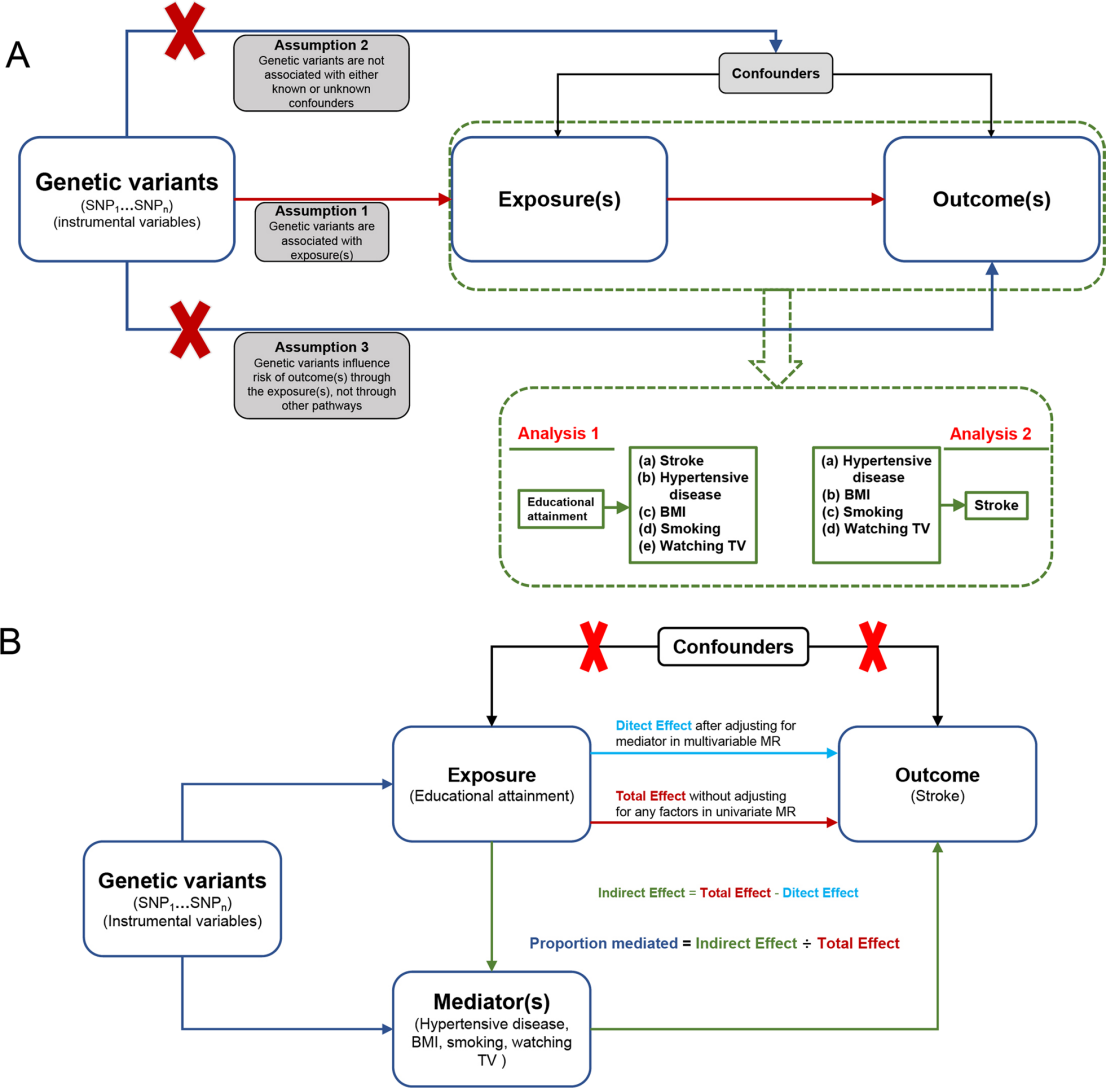
Study design overview. **A** Univariable MR. MR fundamental assumptions: (1) Relevance assumption: The genetic variants (instrumental variables) are strongly correlated with exposure(s) (*P* < 5E−8; *r*^2^ < 0.001 and distance > 10 MB, the SNPs are in pairwise linkage disequilibrium). (2) Independence assumption: no unmeasured confounders of the correlations are present between genetic variants and outcome(s). (3) Exclusion restriction assumption: the genetic variants influence the outcome(s) only via exposure(s). **B** Multivariable MR. MR: Mendelian randomization; PM: Proportion mediated; SNP: single nucleotide polymorphism; BMI: Body mass index; Educational attainment: years of schooling; Smoking: smoking behavior; Watching TV: time spent watching the television

The overall study design flowchart of the MR is displayed in Fig. 1. The GWAS summary level data for years of schooling (an indicator of educational attainment), hypertensive disease, BMI (an indicator of obesity), smoking behavior, time spent on watching TV (an indicator of physical inactivity), and stroke were extracted from the IEU Open GWAS database (https://gwas.mrcieu.ac.uk/). The years of schooling were deemed as exposure. The four modifiable factors (hypertensive disease, BMI, smoking behavior, and time spent on watching TV) were defined as mediators. The risk of stroke was regarded as the outcome. The genetic variants (SNPs) GWAS data were used as instrumental variables to explore the genetically predicted underlying mechanism of the effect of educational attainment on the risk of stroke by mediating hypertensive disease, BMI, smoking behavior, and time spent on watching TV. IVW regression was used to estimate the causality and causal effect size in univariable and multivariable analyses. The proportions mediated (PM) were calculated using the indirect effect size divided by the total effect size and the method described in a previous study [16].

### Data sources

The traits’ GWAS summary data, including years of schooling, hypertensive diseases, BMI, smoking behavior, time spent on watching TV, and stroke, were obtained from extensive sample studies of European ancestry populations. The genetic data related to years of schooling (standard deviation [SD]: 3.71 years) were derived from a GWAS study with 293,723 participants (GWAS ID: ieu-a-1001) [17]. The hypertensive disease-related summary-level data originated from a GWAS study with 55,955 cases and 162,837 controls (GWAS ID: finn-b-I9_HYPERTENSION). The BMI-related genetic data involving 681,275 populations were obtained from a GWAS meta-analysis (GWAS ID: ieu-b-40) [18]. The genetic data on the association with time spent on watching TV association among 437,887 individuals were obtained from a GWAS analysis of the UK Biobank (GWAS ID: ukb-b-5192). The smoking-related GWAS data were derived from 632,802 participants (smoking: 311,629; never smoked: 321,173) (GWAS ID: ieu-b-4877) [19]. The summary association results of the stroke, encompassing all stroke subtypes, were derived from a GWAS meta-analysis of 40,585 stroke cases and 406,111 controls (GWAS ID: ebi-a-GCST005838) [20].

### Statistical analysis

#### Univariable MR analysis

The network relationship among exposure (educational attainment), mediators (hypertensive disease, BMI, smoking behavior, and time spent watching TV), and outcome (stroke) was determined by conducting univariable MR analyses by using the IVW approach. The flowchart of the analyses is exhibited in Fig. 1A. (a) According to a genome-wide significance (*P* < 5E−8) and parameter of pairwise linkage disequilibrium (LD; *r*^2^ < 0.001 and clump window > 10 MB, among SNPs), SNPs that are independently associated with educational attainment, hypertensive disease, BMI, smoking behavior, and time spent on watching TV were identified. To ensure the independence of SNPs, we utilized the PhenoScanner database (http://www.phenoscanner.medschl.cam.ac.uk/) to examine and remove SNPs associated with confounders. Then, the SNPs were used as instrumental variables to perform causal inference by using the IVW method. The F statistic was used to assess the instrument strength, and its calculative method was described in a previous study [21–23]. At F statistic > 10, no weak instrument bias was considered. (b) The causal direction was assessed from exposure (educational attainment) to each mediator (hypertensive disease, BMI, smoking behavior, and time spent watching TV) and outcome (stroke), and the causal effect size was calculated. (c) The causal direction was examined from each mediator (hypertensive disease, BMI, smoking behavior, and time spent watching TV) to outcome (stroke), and the causal effect size was calculated.

The reliance and robustness of the above causal estimation of univariable MR were examined using a series of sensitivity analyses. First, the power of causal estimation was computed using an online tool (https://shiny.cnsgenomics.com/mRnd/) based on a type I error rate of 0.05 [24]. A power of more than 80% was regarded as strong evidence. Then, the consistency of causal direction from the IVW method was examined using four methods, namely, MR-Egger [25], Maximum likelihood [26], MR-pleiotropy residual sum outlier (MR-PRESSO) [27], and robust adjusted profile score (MR-RAPS) [28]. Third, the heterogeneity of the included SNPs was estimated using the Cochran’s Q statistic in the IVW and MR-Egger models. Significant heterogeneity was considered at *P* < 0.05. Fourth, the MR-Egger regression was used to inspect possible pleiotropy. The MR-PRESSO, MR-Egger, and IVW approaches were used to identify and remove potential outliers that can cause underlying pleiotropy. Fifth, the leave-one-out analysis was used to examine whether any single SNP significantly influenced the causal effect of IVW estimation. Finally, the MR Steiger test was used to inspect the correctness of causal evaluation.

#### Multivariable MR analysis

Multivariable MR was used to expound the causal effect of educational attainment on the risk of stroke mediated by the four mediators (hypertensive disease, BMI, smoking behavior, and time spent on watching TV). The diagram of multivariable MR is displayed in Fig. 1B. The effect of educational attainment on the risk of stroke was divided into direct and indirect effects. First, educational attainment and individual mediator were included in multivariate MR for calculating the direct and indirect (mediated by a single mediator) effects of educational influence on the risk of stroke. Next, educational attainment and all mediators were included in multivariate MR for estimating the direct and indirect (mediated by all mediators) effects of educational attainment on the risk of stroke. Mediation analysis was conducted as previously described [16]. The proportion mediated (PM) was computed by dividing the indirect effect by the total effect [16].

All MR analyses were performed in R programming language (version 4.0.2) by using the TwoSampleMR (version 0.5.6) and MR-PRESSO (version 1.0) packages in R.

## Results

### Network relationship among educational attainment, stroke, and four mediators in univariable MR

**Fig. 2:**
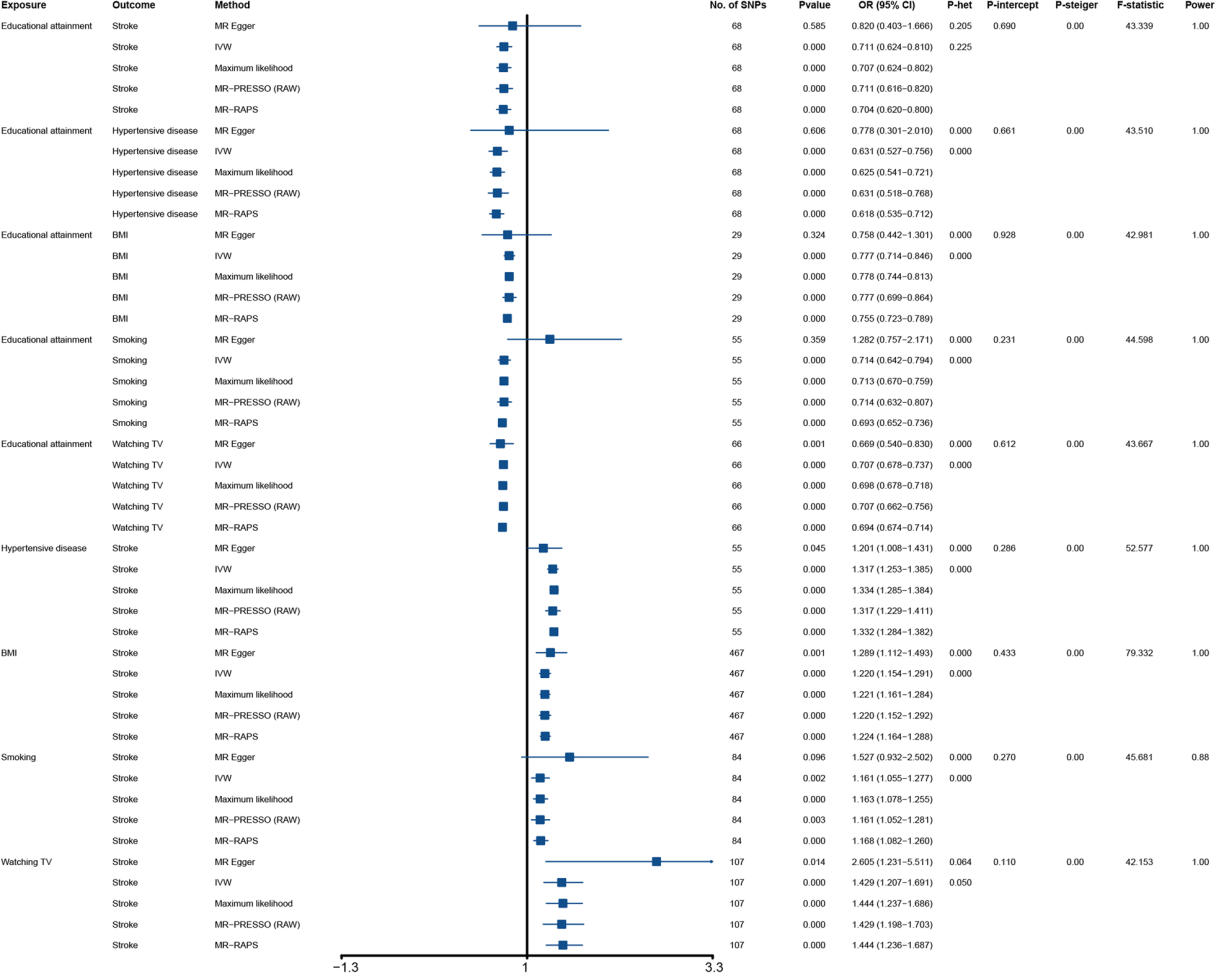
Forest plot displays the results of univariable MR analyses.MR: Mendelian randomization; SNP: single nucleotide polymorphism; BMI: Body mass index; Educational attainment; years of schooling; Smoking: smoking behavior; Watching TV: time spent watching television; IVW: inverse-variance-weighted; MR-PRESSO: MR-pleiotropy residual sum outlier; MR-RAPS: MR-robust adjusted profile score; OR: odds ratio; CI: confidence intervals; *P*-het: *P* value for heterogeneity based on Cochran Q test; *P*-intercept: *P* value for MR-Egger intercept; *P*-Steiger: *P* value for MR-Steiger test

Detailed information of the included SNPs can be found in Tables S1–S9 of Additional file 1. In univariable MR, all SNPs’ F statistics were greater than 10, suggesting no weak-instrument bias. The results from genetically predicted causal association showed that educational attainment was negatively associated with the stroke risk (IVW, odds ratio [OR] = 0.711, 95% confidence interval [CI]: 0.624–0.810, *P* = 3.27E−07, power = 1), hypertensive disease risk (IVW, OR = 0.631, 95% CI 0.527–0.756, *P* = 6.07E–07, power = 1), BMI (IVW, OR = 0.777, 95% CI 0.714–0.846, *P* = 5.87E−09, power = 1), smoking (IVW, OR = 0.714, 95% CI 0.642–0.794, *P* = 4.94E−10, power = 1), and time spent on watching TV (IVW, OR = 0.707, 95% CI 0.678–0.737, *P* = 7.51E–60, power = 1; Fig. 2). Based on the results of the causal association among the four modifiable mediators and the risk of stroke, that the genetically predicted hypertensive disease, BMI, smoking, and time spent on watching TV were positively associated with the risk of stroke, and with the ORs were 1.317 (95% CI 1.253–1.385, *P* = 3.97E−27, power = 1), 1.220 (95% CI 1.154–1.291, *P* = 3.30E−12, power = 1), and 1.429 (95% CI 1.207–1.961, *P* = 3.47E−05, power = 0.880; Fig. 2).

**Fig. 3:**
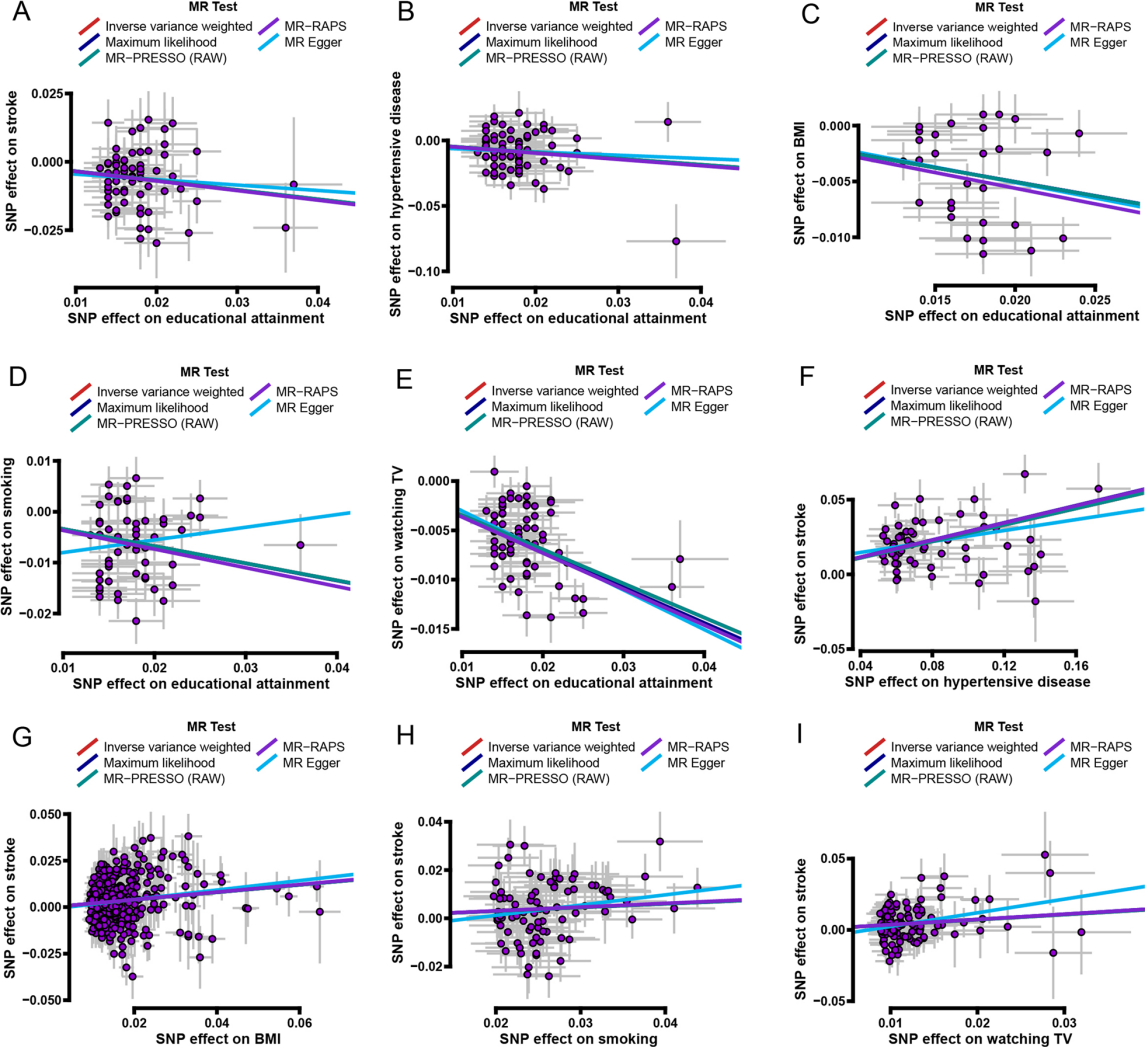
Scatter plots showing the estimated causal associations with five methods. **A** Causal association between educational attainment and the risk of stroke. **B** Causal association between educational attainment and the risk of hypertensive disease. **C** Causal association between educational attainment and BMI. **D** Causal association between educational attainment and smoking. **E** Causal association between educational attainment and time spent watching TV. **F** Causal association between hypertensive disease and the risk of stroke. **G** Causal association between BMI and the risk of stroke. **H** Causal association between smoking and the risk of stroke. **I** Causal association between time spent watching TV and the risk of stroke. MR: Mendelian randomization; SNP: single nucleotide polymorphism; BMI: Body mass index; Educational attainment: years of schooling; Smoking: smoking behavior; Watching TV: time spent watching the television; IVW: inverse-variance-weighted; MR-PRESSO: MR-pleiotropy residual sum outlier; MR-RAPS: MR-robust adjusted profile score

**Fig. 4:**
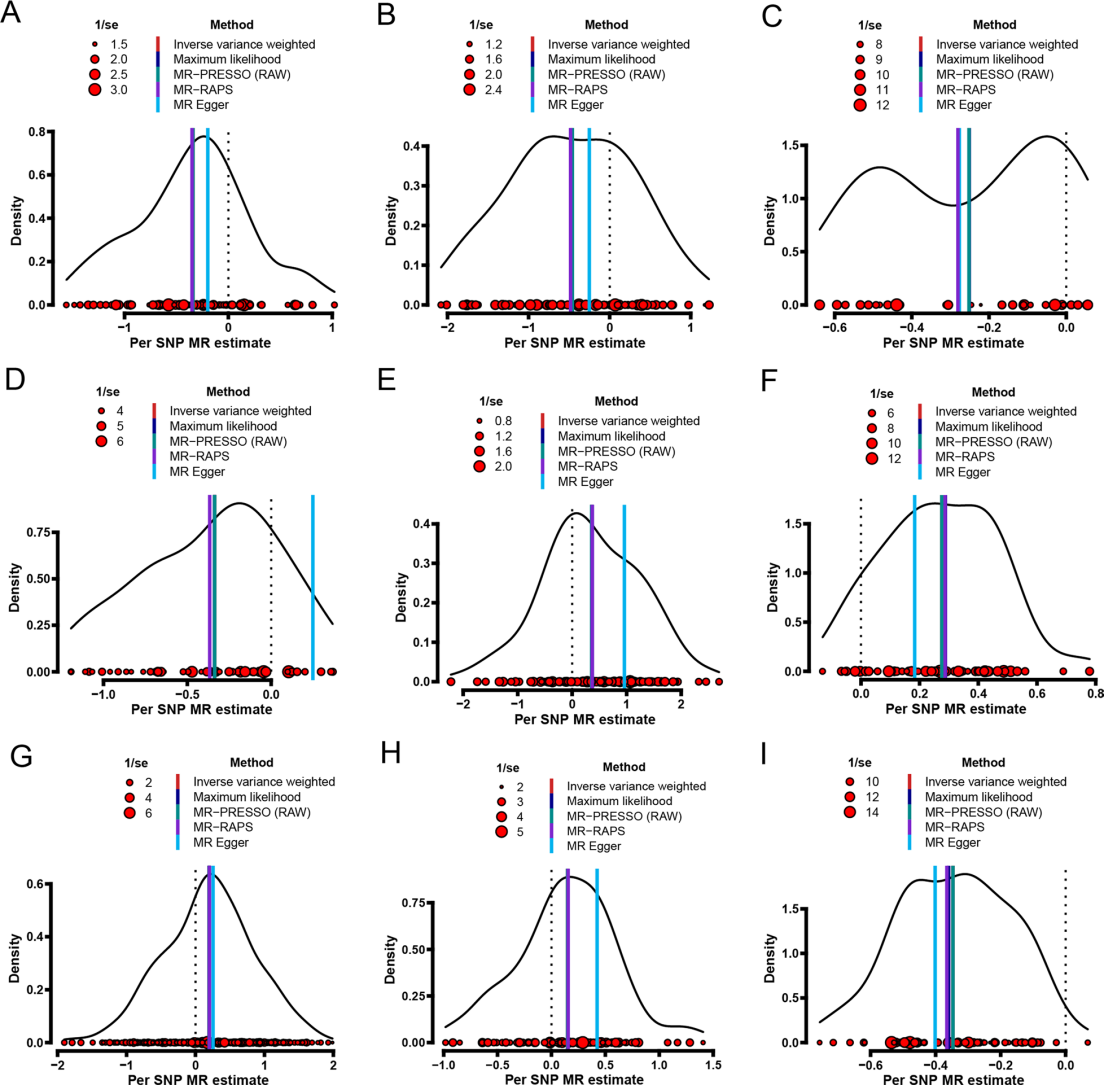
Density plots showing that the five methods estimate the effect of SNPs on the outcome of interest. **A** Effect of educational attainment-related SNPs on the risk of stroke. **B** Effect of educational attainment-related SNPs on the risk of hypertensive disease. **C** Effect of educational attainment-related SNPs on BMI. **D** Effect of educational attainment-related SNPs on smoking. **E** Effect of educational attainment-related SNPs on time spent on watching the TV. **F** Effect of hypertensive disease-related SNPs on the risk of stroke. **G** Effect of BMI-related SNPs on the risk of stroke. **H** Effect of smoking-related SNPs on the risk of stroke. **I** Effect of time spent on watching the TV-related SNPs on the risk of stroke. MR: Mendelian randomization; SNP: single nucleotide polymorphism; IVW: inverse-variance-weighted; MR-PRESSO: MR-pleiotropy residual sum outlier; MR-RAPS: MR-robust adjusted profile score

A series of sensitivity analyses was conducted to test the robustness and reliance of the above univariable MR analyses. First, MR-Egger, Maximum likelihood, MR-PRESSO, and MR-RAPS were used to validate the stability of IVW causal estimation, and the results showed that their causal estimations were almost consistent with the IVW method (Figs. 3 and 4). Second, the heterogeneity of the included SNPs was examined using Cochran’s Q statistic in IVW and MR-Egger model. The results indicated certain heterogeneity among the included SNPs in most univariable MR analyses (Fig. 2). The heterogeneities may be derived from SNP influence on exposures differently. Third, the pleiotropy of the included SNPs was assessed using the MR-Egger method. The results showed that all included SNPs had no pleiotropy (all *P*_-intercept_ > 0.05) (Fig. 2). Fourth, leave-one-out analysis was used to evaluate the stability of IVW causal estimations. The result showed that no single SNP significantly turned IVW causal estimation in the univariable MR analyses (all *P* < 0.05, Tables S10–S18 in Additional file 2). Finally, the MR-Steiger directionality test was used to examine whether the causal direction of IVW estimation is correct. The results showed that all IVW causal estimations were accurate in the univariable MR analyses (all *P*_-Steiger_ < 0.05, Fig. 2).

### Multivariable MR and mediation analyses

**Fig. 5:**
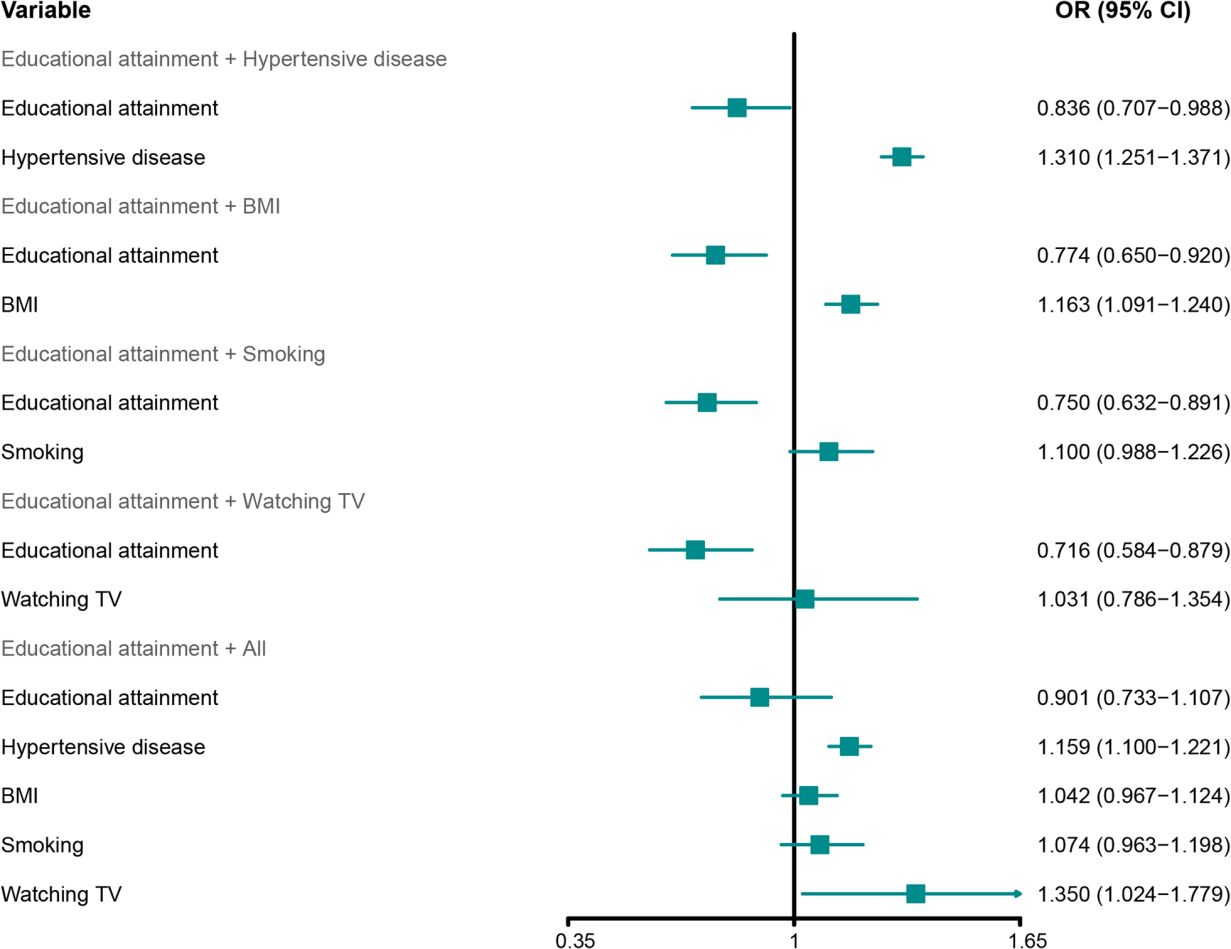
Forest plot denotes the results of multivariable MR analyses.MR: Mendelian randomization; BMI: Body mass index; Educational attainment: years of schooling; Smoking: smoking behavior; Watching TV: time spent watching television; IVW: inverse-variance-weighted; MR-PRESSO: MR-pleiotropy residual sum outlier; MR-RAPS: MR-robust adjusted profile score; OR: odds ratio; CI: confidence intervals

To further elucidate the effect of educational attainment on the risk of stroke mediated by the four modifiable mediators, we performed multivariable MR and mediation analyses. The total effect consists of direct and indirect (mediating) effects. The total effect was obtained from the causal effect estimation of the influence of educational attainment on stroke risk based on univariable MR analysis. The direct and indirect (mediating) effects of educational attainment on stroke risk could be calculated by adjusting for other factors of interest by using multivariable MR. Individual mediators were included for calculating the direct and indirect (mediating) effects of education on stroke risk. After controlling for hypertensive disease, the effect of educational attainment on stroke risk had an OR of 0.836 (95% CI 0.707–0.988; Fig. 5). The PM of hypertensive disease was 47.35%. After adjustment for BMI, the effect of educational attainment on stroke risk had an OR of 0.774 (95% CI 0.650–0.920; Fig. 5); The PM of BMI was 24.74%. After correcting for smoking behavior, the effect of educational attainment on stroke risk had an OR of 0.750 (95% CI 0.632–0.891; Fig. 5); The PM of smoking behavior was 15.72%. After correcting for time spent on watching TV, the effect of educational attainment on stroke risk had an OR of 0.716 (95% CI 0.584–0.879; Fig. 5); The PM of time spent on watching TV was 2.29%. In combination with the four mediators for adjustment, the effect of educational attainment on stroke risk had an OR of 0.901 (95% CI 0.733–1.107; Fig. 5). The PM of the four combined mediators was 69.32%.

## Discussion

In the present work, large-scale GWAS summary data were used to investigate the genetically predicted causal association between educational attainment, hypertensive disease, BMI, smoking behavior, time spent on watching TV, and stroke risk by using the MR method. Educational attainment, hypertensive disease, BMI, smoking behavior, and watching TV were causally associated with stroke risk. Furthermore, the effect of educational attainment on the risk of stroke was mediated by the four modifiable factors (hypertensive disease, BMI, smoking behavior, and time spent on watching TV).

The harm of stroke in the global population is grievous, and it has become the second-leading cause of death in global populations [29]. The morbidity of stroke is significantly different in people with another educational attainment. Individuals with high educational level have a low stroke incidence [30]. A recent meta-analysis that included 90 studies involving more than 160,000 stroke cases and 5 million controls indicated that people with lower educational level had a higher risk of stroke than those with a higher educational level (OR = 1.35, 95% CI 1.24–1.48) [31]. Furthermore, Wen et al. recently conducted an MR study that included 11,509 individuals and found a negative causal association between educational attainment and the risk of stroke [32]. The results of the present work also suggested that genetically predicted lower educational attainment was causally associated with higher stroke risk (OR = 0.711, 95% CI 0.624–0.810). However, some differences were observed between the present work and that of Wen et al. First, the present work involves a two-sample MR investigation and included large-scale GWAS data with more than 700,000 individuals. Second, our results are supported by more statistical evidence from multiple sensitivity analyses. Third, multivariable MR analyses were carried out to correct for confounding factors and validate causal inference’s stability. Finally, mediation analyses were carried out to explain the underlying mechanism in which educational attainment influences the risk of stroke.

Hypertension is a key risk factor for stroke [33–35]. A cohort study followed up approximately 80,000 participants for more than 10 years and found that hypertensive participants have a higher incidence of stroke than those without hypertension [36]. Similarly, the present work indicated that genetically predicted hypertensive disease is a causal risk factor for stroke. The incidence of hypertensive disease in populations with different educational levels has been found divergent [37–39]. Individuals with higher educational attainment have a lower risk of hypertensive disease [40, 41]. The results of the present study showed that genetically predicted higher educational attainment was causally associated with a lower risk of hypertensive disease. Furthermore, the effect of educational attainment on the risk of stroke was mediated by hypertensive disease with PM of 47.35%. Based on the above evidence, controlling hypertension can partly attenuate the stroke risk caused by educational inequality.

Obesity is a highly prevalent disease and is closely related to the risk of stroke [42–45]. BMI remains as a primary indicator of obesity in clinical work. A large meta-analysis that included 97 prospective studies involving 1.8 million individuals revealed that an increase of 5 kg/m^2^ in BMI is associated with a higher stroke risk with an HR of 1.18 (95% CI 1.14–1.22) after adjustment for confounders. Similarly, in the present work, genetically determined increase in BMI was causally related to a higher risk of stroke. In addition, observational data showed that the incidence of obesity in individuals with higher educational attainment was lower than in those with lower educational attainment [46]. In the present work, genetically predicted higher educational attainment can attenuate the risk of obesity. However, whether obesity mediates the causal association between educational attainment and stroke risk remains unexplained. Moreover, the effect of educational attainment on the risk of stroke was mediated by obesity, accounting for 24.74%. Therefore, reasonable weight control in a healthy way may effectively attenuate the risk of stroke caused by lower educational attainment.

Smoking and low physical activity were all unhealthy lifestyles associated with an increased risk of stroke [29, 47, 48]. The long time spent on watching TV is a critical indicator of low physical activity. In the univariable MR, smoking behavior and time on spent watching TV were causally associated with the risk of stroke. In addition, genetically predicted higher educational attainment was negatively associated with smoking and time spent watching TV. Therefore, individuals with high educational attainment have a more strengthened health consciousness [49]. Moreover, the findings from mediation analysis indicated that smoking and time spent on watching TV were all mediators in linking the causal relationship between educational attainment and the risk of stroke. Strengthening health education against smoking and increasing physical activity are beneficial for weakening the bad effect of educational inequality on stroke risk.

Considering the importance of the four modifiable factors in mediating the causal link between educational attainment and the risk of stroke, the combined PM of the four mediators was calculated. When all four mediators were included for conducting a mediation analysis, the PM reached 69.32%. Based on the above evidence, stroke risk caused by educational inequality can be attenuated by reducing risks for hypertension and obesity, stopping smoking, and increasing physical activity.

The present work has some strengths. On the one hand, GWAS summary-level data from recent extensive sample studies were used, and the included SNPs were more comprehensive and reliable. On the other hand, the reliance and stability of the causal inferences from our work were supported by statistical evidence from multiple sensitivity analyses.

However, several limitations should not be overlooked. First, although the four modifiable factors largely explain the effect of educational attainment on the risk of stroke, the remaining part effect still needs further exploration. This way can be used to completely blockade educational inequality’s effect on stroke risk. Second, the GWAS summary data were derived from European populations, and the findings may have some potential bias if generalized to non-European ancestry. Third, considering the limitations in the GWAS summary data, a stratified analysis of demographic characteristics, such as gender and age, was not achieved.

In conclusion, by using extensive genetic data, MR analyses were conducted to clarify the causal association among educational attainment, four modifiable factors (hypertension, BMI, smoking, and time spent on watching TV), and the risk of stroke. Educational attainment and the four modifiable factors were all causally associated with the risk of stroke. Over half of the effect of educational attainment on the risk of stroke was mediated by the four modifiable factors. The findings provide new insight into drawing up public policies to block the stroke risk caused by educational inequality.

## Supplementary Information

**Additional file 1: Table S1.** The detailed information on single-nucleotide polymorphisms for estimating the causal association between years of schooling and the risk of stroke. **Table S2.** The detailed information on single-nucleotide polymorphisms for estimating the causal association between years of schooling and the risk of hypertensive disease. **Table S3.** The detailed information on single-nucleotide polymorphisms for estimating the causal association between years of schooling and the risk of obesity. **Table S4.** The detailed information on single-nucleotide polymorphisms for estimating the causal association between years of schooling and smoking. **Table S5.** The detailed information on single-nucleotide polymorphisms for estimating the causal association between years of schooling and time spent. **Table S6.** The detailed information on single-nucleotide polymorphisms for estimating the causal association between hypertensive disease and the risk of stroke. **Table S7.** The detailed information on single-nucleotide polymorphisms for estimating the causal association between body mass index and the risk of stroke. **Table S8.** The detailed information on single-nucleotide polymorphisms for estimating the causal association between smoking and the risk of stroke. **Table S9.** The detailed information on single-nucleotide polymorphisms for estimating the causal association between time spent watching television and the risk of stroke.

**Additional file 2: Table S10.** Leave-one-out analysis of the causal relationship between years of schooling and the risk of stroke. **Table S11.** Leave-one-out analysis of the causal relationship between years of schooling and the risk of hypertensive disease. **Table S12.** Leave-one-out analysis of the causal relationship between years of schooling and the risk of obesity. **Table S13.** Leave-one-out analysis of the causal relationship between years of schooling and smoking. **Table S14.** Leave-one-out analysis of the causal relationship between years of schooling and time spent watching television. **Table S15.** Leave-one-out analysis of the causal relationship between hypertensive disease and the risk of stroke. **Table S16**. Leave-one-out analysis of the causal relationship between obesity and the risk of stroke. **Table S17.** Leave-one-out analysis of the causal relationship between smoking and the risk of stroke. **Table S18.** Leave-one-out analysis of the causal relationship between time spent watching television and the risk of stroke

## Data Availability

The datasets generated for this study are available on request to the corresponding author.

## Abbreviations

MR: Mendelian randomization
BMI: Body mass index
TV: Television
OR: Odds ratio
CI: Confidence intervals
PM: Proportion mediated
SNPs: Single-nucleotide polymorphisms
GWAS: Genome-wide association study
IVW: Inverse-variance-weighted
MR-PRESSO: MR-pleiotropy residual sum outlier
MR-RAPS: MR-robust adjusted profile score
